# The significance of genetic mutations and their prognostic impact on patients with incidental finding of isolated del(20q) in bone marrow without morphologic evidence of a myeloid neoplasm

**DOI:** 10.1038/s41408-020-0275-8

**Published:** 2020-01-23

**Authors:** Aishwarya Ravindran, Rong He, Rhett P. Ketterling, Majd D. Jawad, Dong Chen, Jennifer L. Oliveira, Phuong L. Nguyen, David S. Viswanatha, Kaaren K. Reichard, James D. Hoyer, Ronald S. Go, Min Shi

**Affiliations:** 1 Division of Hematopathology, Department of Laboratory Medicine and Pathology, Mayo Clinic, Rochester, MN USA; 2 Division of Laboratory Genetics and Genomics, Mayo Clinic, Rochester, MN USA; 3 Division of Hematology, Mayo Clinic, Rochester, MN USA

**Keywords:** Cancer genetics, Haematological cancer

## Abstract

Patients with a sole del(20q) chromosomal abnormality and without morphologic features of a myeloid neoplasm (MN) have shown variable clinical outcomes. To explore the potential risk stratification markers in this group of patients, we evaluated their genetic mutational landscape by a 35-gene MN-focused next-generation sequencing (NGS) panel and examined the association of mutations to progression of MNs. Our study included 56 patients over a 10-year period with isolated del(20q), of whom 23 (41.1%) harbored at least one mutation. With a median follow-up of 32.6 months (range: 0.1−159.1), 9 of 23 patients with mutation(s) progressed to MNs, while all 33 patients without mutations did not progress to MN. Kaplan−Meier survival analysis demonstrated the presence of mutation(s) as a significant risk factor for progression to MN (*P* *<* 0.0001). MN progression was strongly associated with the presence of non-*DNMT3A/TET2/ASXL1* epigenetic modifiers and nonspliceosome mutations (*P* *=* 0.003). There was no significant difference among patients with and without MN progression with respect to the number of mutations, variant allele frequency, percentage of del(20q), and other clinical/laboratory variables. This study illustrates the underlying genetic heterogeneity and complexity of isolated del(20q), and underscores the prognostic value of NGS mutational analysis in these cases.

## Introduction

Deletion of the long arm of chromosome 20q [del(20q)] is a recurring chromosomal abnormality identified in a variety of myeloid neoplasms (MNs), including myelodysplastic syndrome (MDS), myeloproliferative neoplasm (MPN), MDS/MPN, and acute myeloid leukemia (AML). Del(20q) may occur as a sole abnormality or in the setting of other cytogenetic alterations, either as an early or late event in patients with MNs. Irrespective of the variable MN subcategories, bone marrow samples from MN patients with del(20q) characteristically show morphologic abnormalities in erythroid precursors and megakaryocytes^1^, including the unique features of neutrophilic erythrophagocytosis and prominent megakaryocytic emperipolesis in MDS^2,3^.

Del(20q) as a sole chromosomal abnormality identified in a bone marrow specimen, which shows no morphologic diagnostic features of MNs (referred to isolated del(20q) in this study), may be incidentally encountered in patients evaluated for nonmyeloid malignancies or unexplained cytopenia(s). According to the WHO classification (fourth edition)^4^, isolated del(20q) is not considered a definitive evidence for MDS in patients with unexplained cytopenia in the absence of morphologic evidence for MDS^5^, which sometimes results in a diagnostic and therapeutic dilemma. Studies have shown variable clinical outcomes in patients with isolated del(20q) and about 10−25% of patients ultimately evolve into various MNs including AML^6,7^. However, the underlying pathogenic mechanisms associated with MN progression are largely unknown in isolated del(20q) patients. In this study, we investigated the mutational landscape in this patient cohort and explored its association to progression into MN(s).

## Methods

### Case selection

**Fig. 1:**
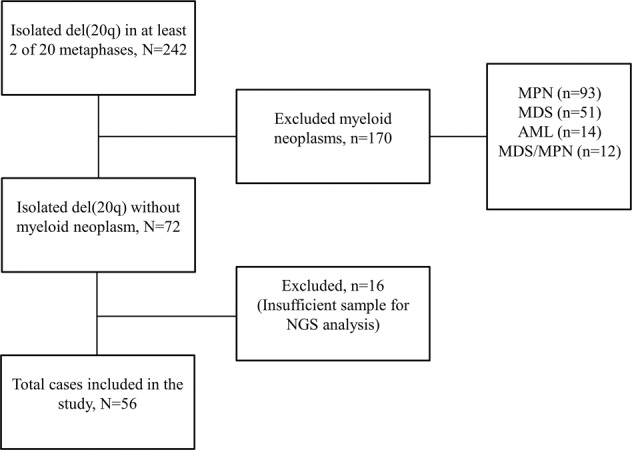
Flow chart depicting selection of patient cohort with isolated del(20q). BM bone marrow, MPN myeloproliferative neoplasm, MDS myelodysplastic syndrome, AML acute myeloid leukemia, NGS next-generation sequencing.

We performed a 10-year retrospective review (January 1, 2005 to September 30, 2015) of the Mayo Clinic cytogenetic database and found 4428 Mayo Clinic patients with cytogenetic abnormalities in bone marrow karyotype analysis. Among these patients, a total of 242 had sole del(20q) observed in at least 2 of 20 metaphases. Bone marrow pathology reports were reviewed and indicated 72 patients had no morphologic diagnostic features of involvement by an MN, thus meeting the inclusion criteria of isolated del(20q) for this study. The remaining 170 patients fulfilled the criteria for MPN (93), MDS (51), AML (14), and MDS/MPN (12) and were excluded. Archived bone marrow aspirate cell pellets with sufficient material for next-generation sequencing (NGS) analysis were available in 56 of 72 cases (Fig. 1). Clinical information was obtained from medical charts for the final study cohort of 56 patients. Progression to MN was determined based on deteriorating clinical/laboratory findings and subsequent confirmation by a bone marrow biopsy. This study was approved by the Mayo Clinic Institutional Review Board.

### Conventional chromosome analysis

Cells from the bone marrow aspirate were cultured (unstimulated) for 24 and 48 h, harvested and G-banded using standard cytogenetic techniques. A total of at least 20 metaphases were fully analyzed and reviewed for each sample when available. A del(20q) was identified in at least 2 of 20 metaphases to be considered a clone.

### Myeloid neoplasm-focused NGS test

DNA was extracted from the available bone marrow cytogenetic cell pellets stored in 3:1 methanol:acetic acid fixative from the 56 patients using Qiagen EZI (Qiagen, Germantown, MD). NGS testing was performed using a targeted OncoHeme panel, which interrogated 35 genes recurrently mutated in MNs, including *ASXL1, BCOR, BRAF, CALR, CBL, CEBPA, CSF3R, DNMT3A, ETV6, EZH2, FLT3, GATA1, GATA2, IDH1, IDH2, JAK2, KIT, KRAS, MPL, MYD88, NOTCH1, NPM1, NRAS, PHF6, PTPN11, RUNX1, SETBP1, SF3B1, SRSF2, TERT, TET2, TP53, U2AF1, WT1*, and *ZRSR2*. Two-hundred nanogram sheared DNA was target-enriched with a custom hybridization-capture reagent (SureSelect^XT^, Agilent, Santa Clara, CA) and sequenced on the MiSeq or HiSeq platforms (Illumina, San Diego, CA) at the Mayo Clinic Clinical Genome Sequencing Laboratory. NGS data were processed through a proprietary bioinformatics analysis pipeline (Mayo NGS Workbench) and genetic variants were classified and annotated in our clinical Molecular Hematopathology laboratory following the American College of Medical Genetics and Genomics (ACMG) five-tier system, the ACMG standards and guidelines for the interpretation of sequencing variants, and the Association for Molecular Pathology (AMP)/ACMG approach to somatic mutation characterization^8^.

### Statistical methods

Statistical analyses were performed using JMP Pro software version 14 (SAS Institute Inc., Cary, NC). Continuous variables are reported as median (range) or mean (standard deviation (SD); range) and categorical variables as number (percentage). When the normality assumption was violated, we used nonparametric tests such as the Fisher’s exact test, chi-square test, or Wilcoxon rank-sum/Mann−Whitney *U* test, as appropriate, for test variables. Statistical significance was based on a two-sided significance level of 0.05. The main end point of the study was progression to a myeloid neoplasm. The median follow-up was calculated from the time of isolated del(20q) detection to progression to MN or last available clinical follow-up for those without progression to MN. The overall survival and time to progression were analyzed using Kaplan−Meier survival curves to account for differences in follow-up per patient, and group comparisons were performed using log-rank tests.

## Results

Fifty-six patients with isolated del(20q) were included in this study, with 42 (75%) male and 14 (25%) female patients. The average age at isolated del(20q) detection was 67.9 years (SD ± 11.0; range: 44−90). At initial diagnosis, the average percentage of del(20q) was 38.5% (SD ± 29.2). The mean hemoglobin (Hb), absolute neutrophil count (ANC), and platelet count (Plt) were 11.7 g/dL (SD ± 1.7; range: 8.3−15.1), 3.2 × 10^9^/L (SD ± 1.8; range: 0.6−9.4) and 149 × 10^9^/L (SD ± 84.4; range: 16−392), respectively. The indications for bone marrow examination were variable and included evaluation for cytopenia(s) (*n* = 17), monoclonal gammopathy (*n* = 3), amyloidosis (*n* = 2), allogeneic stem-cell transplant donor evaluation (*n* = 1), and staging for multiple myeloma (*n* = 17), non-Hodgkin lymphoma (*n* = 14), and chronic lymphocytic leukemia (CLL, *n* = 2). At a median follow-up of 32.6 months (range: 0.1−159.1), nine progressed to a myeloid neoplasm. The clinical and laboratory features of patients with and without progression are described in Table 1.

**Table 1:** Comparison of clinical and laboratory features of patients with isolated del(20q) among those with progression versus without progression to a myeloid neoplasm.

Variable	Progression to MN (*n* = 9)	No progression to MN (*n* = 47)	*P* value
Age, years	Mean: 69.4 (SD ± 9.8) (range: 55−88)	Mean: 67.6 (SD ± 11.3) (range: 44−90)	0.66
Sex	Male: 6 (66.7%) Female: 3 (33.3%)	Male: 36 (76.6%) Female: 11 (23.4%)	0.68
Hemoglobin, g/dL	Mean: 12.3 (SD ± 1.6) (range: 9.3−14.7)	Mean: 11.5 (SD ± 1.7) (range: 8.3−15.1)	0.20
Absolute neutrophil count, ×10^9^/L	Mean: 3.2 (SD ± 2.5) (range: 0.6−8)	Mean: 3.1 (SD ± 1.6) (range: 0.8−9.4)	0.88
Platelet count, ×10^9^/L	Mean: 156.9 (SD ± 108.7) (range: 61−392)	Mean: 147 (SD ± 80.3) (range: 16−385)	0.75
% Del(20q)	Mean: 30.6 (SD ± 29.2) (range: 10−100)	Mean: 39.9 (SD ± 29.3) (range: 6.7−100)	0.39
Cytotoxic chemotherapy	7 (77.8%)	31 (65.9%)	0.70
Gene mutations ≥ 1	9 (100%) (1 Mut: *n* = 5; 2 Mut: *n* = 1; 3 Mut: *n* = 3)	14 (29.8%) (1 Mut: *n* = 10; 2 Mut: *n* = 1; 3 Mut: *n* = 3)	0.0001

*MN* myeloid neoplasm, *Mut* mutation.

**Fig. 2:**
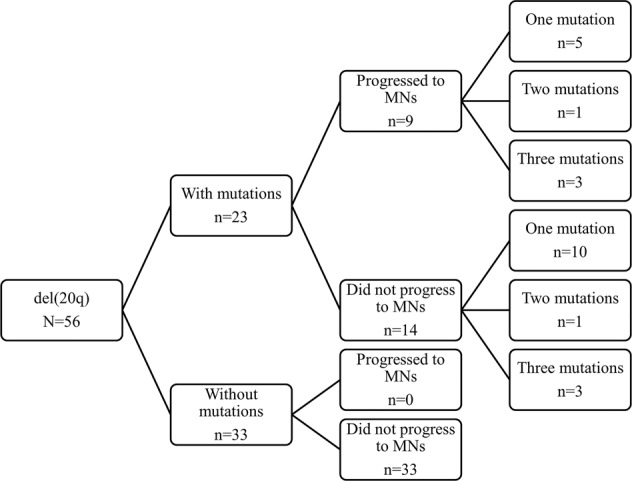
Summary of pathogenic mutations and clinical outcomes of patients with isolated del(20q). MNs myeloid neoplasms.

At the time of isolated del(20q) detection, NGS revealed mutations in 23 of the 56 (41.1%) patients, while the remaining 33 (58.9%) patients did not show mutations (Fig. 2). Mutations were detected in the following genes: *TET2 (8), ASXL1 (7), SRSF2 (3), SF3B1 (3), DNMT3A (3), PHF6 (2), CBL (2), U2AF1 (2), IDH1 (1), IDH2 (1), BCOR (1), JAK2 (1), PTPN11 (1), TP53 (1)*, and *RUNX1 (1)*. The variant allele fraction (VAF) of mutated genes ranged from 5.2 to 53.4%. Overall, 15 patients harbored one mutation, two patients harbored two mutations, six patients harbored three mutations, and no patient showed more than three mutations (Fig. 2).

Nine of the 23 (39.1%) patients with mutation(s) progressed to an MN including five MDS, two AML, and two MDS/MPN unclassifiable, at a median follow-up of 36.7 months (range: 1.9−57.4). At the time of isolated del(20q), among these nine patients, five had one mutation (*BCOR, TP53*, *DNM3TA, CBL, IDH2*) with VAF ranging from 5.2 to 53.4%, one had two mutations (*SF3B1/CBL)* each with VAF of 25.3%, and three had three mutations (*PHF6/TET2/TET2, ASXL1/IDH1/SRSF2, DNM3TA/PTPN11/TET2*) with VAF ranging from 24.7 to 46.4% (Table 2). Five of the nine patients had subsequent NGS performed at the time of MN progression (Table 2, patients #1−4, and #7). Four patients (#1, 2, 4, 7) acquired additional mutations during disease progression; two of these four patients (#1, 4) also acquired additional cytogenetic abnormalities. One patient (#3) did not acquire additional mutations, but the VAF of the pre-existing mutation(s) slightly increased over the interval. Patients #5 and #9 did not have NGS performed at progression; however, they acquired complex cytogenetics around the time of progression. Interestingly, del(20q) disappeared in two patients (#5, 7) and decreased in four patients (#1, 2, 3, 9) during disease progression. Among these six patients who had disappearance/decrease of del(20q) during disease progression, some gained another complex clone (patients #1, 5, 9), some obtained an additional mutation in a different gene (patients #2, 7), one had an increase in the allele burden of the existing mutation (patient #3) (Table 2). These findings indicated that the del(20q) clone had survival/proliferation disadvantage in comparison to other aggressive clones.

**Table 2:** Clinicopathologic features and genetic mutations in isolated del(20q) patients who progressed to myeloid neoplasms.

Pt#			At initial isolated del(20q) detection				At progression				
	Age/Sex	Indication	Bone marrow morphology	Mutations detected; (VAF)	Cytogenetics	Myeloid neoplasm progressed	Bone marrow morphology	Mutations detected; (VAF)	Cytogenetics	Time to progression (months)	Time to final follow-up (months)
1	75/M	Staging for primary diffuse large B-cell lymphoma of the CNS	No lymphoma present; Occasional large megakaryocytes	***TP53***: c.658 T > A; p.Tyr220Asn; (5.2%)	46,XY,del(20)(q11.2q13.3)[10]/46,XY[10]	AML, likely therapy-related	80% blasts; occasional hypogranular neutrophils; No lymphoma present	***TP53***: c.713 G > T;p.Cys238Phe; (10.1%)	42-43,X,-Y,add(5)(q11.2),-6,-7,add(8)(q22),del(9)(q13q22),add(16)(q22), i(17)(q10),-18, +r[3], +mar[10][cp16]/42,sl,add(11)(q23),add(21)(p11.2)[2]/ 46,XY,del(20)(q11.2q13.3)[2]	32.8	33.5
2	70/M	Hairy cell leukemia	Hairy cell leukemia (80−90%)	***CBL*:** c.1096-1 G > C; p.? (25.3%) ***SF3B1***: c.1998G > C; p.Lys666Asn (25.3%) ***BRAF***: c.1799T > A; p.Val600Glu (12.8%)^a^	46,XX,del(20)(q11.2q13.3)[20]	MDS/MPN, unclassifiable	Hairy cell leukemia (10−20%); Hyperlobate and hyperchromatic megakaryocytes forming clusters; ring sideroblasts (10−15%)	***CBL***: c.1096-1 G > C; p.? (32%) ***SF3B1***: c.1998G > C; p.Lys666Asn; (37%) ***EZH2***: c.1481del; p.Pro494Glnfs*20 (28%)	46,XY,del(20)(q11.2q13.3)[9]/46,XY[6]	40.2	99.0
3	70/M	Pancytopenia	Rare small, monolobate megakaryocytes	***ASXL1***: c.3119del;p.Ser1040Leufs*7 (12.8%) ***IDH1***: c.394 C > T;p.Arg132Cys (32.2%) ***SRSF2***: c.284 C > T;p.Pro95Leu (29.1%)	46,XY,del(20)(q11.2q13.3)[2]/46,XY[18]	MDS with excess blasts-1	Trilineage dysplasia and 8% blasts	***ASXL1***: c.3119del;p.Ser1040Leufs*7; (11%) ***IDH1***: c.394 C > T;p.Arg132Cys (35%) ***SRSF2***: c.284 C > T;p.Pro95Leu; (31%)	46,XY,del(20)(q11.2q13.3)[1]/46,XY[19]	1.9	88.0
4	73/F	Chronic neutropenia, thrombocytopenia	Rare hypogranular neutrophils; rare hypolobate/bilobate megakaryocytes	***PHF6***: c.941 T > C; p.Ile314Thr; (10%) ***TET2***: c.3818 G > C; p.Cys1273Ser; (44.8%) ***TET2***: c.4011 T > G; p.Tyr1337*; (42.7%)	46,XX,del(20)(q11.2q13.3)[2]/46,XX[18]	MDS w/multilineage dysplasia	Trilineage dysplasia	***PHF6***: c.941 T > C; p.Ile314Thr; (16%) ***TET2***: c.3818 G > C; p.Cys1273Ser; (48.2%) ***TET2***: c.4011 T > G; p.Tyr1337*; (49.1%) ***WT1***: probable translocation, not further classifiable (6%)	46,XX,ider(20)(p11.1)del(20)(q11.2q13.3)[12]/46,XX,del(20)(q11.2q13.3)[5]/46,XX[3]	53.9	54.3
5	58/M	Multiple myeloma follow-up	No plasma cell neoplasm; rare atypical small megakaryocytes and <1% ringed sideroblasts	***DNMT3A***: c.2645 G > A; p.Arg882His; (10.7%)	46,XY,del(20)(q11.2q13.1)[8]/46,XY[12]	MDS with excess blasts-1	Plasma cell neoplasm (50−60%); mild erythroid dysplasia megakaryocytic dysplasia and 7% blasts	ND	45,XY,-3,i(4)(q10),add(5)(q11.2)[cp11]/46,idem, +mar[8]/46,XY[1]	57.4	64.3
6	65/F	Multiple myeloma follow-up	No plasma cell neoplasm; rare atypical megakaryocytes with large and hyperlobate nuclei	***IDH2***: c.419 G > A; p.Arg140Gln; (12.1%)	46,XX,del(20)(q11.2q13.3)[3]/46,XX[17]	MDS, unclassifiable	Plasma cell neoplasm (20−30%) dysmegakaryopoiesis	ND	46,XX,del(20)(q11.2q13.3)[4]/46,XX[16]	36.7	45.4
7	88/M	Idiopathic immune thrombocytopenia	Normal marrow	***BCOR***: c.4493dup;p.Tyr1498*; (53.4%)	46,XY,del(20)(q11.2q13.1)[2]/46,XY[18]	AML	25% marrow blasts; 4% circulating blasts.	***BCOR***: c.4493dup;p.Tyr1498*; (55%) ***PHF6***: c.673 C > T; p.Arg225*; (22%)	46. XY [20]	50.6	56.9
8	74/F	CLL with thrombocytopenia	No CLL present; Slightly hypercellular marrow.	***CBL***: c.1151 G > A;p.Cys384Tyr; (46.8%)	46,XX,del(20)(q11.2q13.3)[20]	MDS-unclassifiable	NA^b^	ND	ND	3.5	5.8
9	55/M	Multiple myeloma follow-up	No plasma cell neoplasm present; Slightly hypercellular bone marrow	***DNMT3A***: c.2662del;p.Leu888Cysfs*18; (44.9%) ***PTPN11***: c.1546 A > G; p.Met516Val; (48.5%) ***TET2***: c.4151 A > G; p.Asp1384Gly; (45.7%)	46,XY,del(20)(q11.2q13.1)[6]/46,XY[15]	MDS/MPN, unclassifiable	No plasma cell neoplasm present; Hypercellular (70%) marrow with dyserythropoiesis, dysmegakaryopoiesis with numerous large atypical forms forming loose clusters	ND	46,XY,del(20)(q11.2q13.1)[5]/46,idem,add(12)(p11.2)[11]/46,XY,t(1;19)(q12;p13.3)[3]/46,XY[1]	32.4	46.9

*AML* acute myeloid leukemia, *CLL* chronic lymphocytic leukemia, *CMN* chronic myeloid neoplasm, *CNS* central nervous system, *ND* not done, *NHL* non-Hodgkin lymphoma, *MDS* myelodysplastic syndrome, *NA* not available, *VAF* variant allele frequency, *VUS* variant of unknown significance.

^a^Patient #2 *BRAF V600E* mutation was attributable to the patient’s known hairy cell leukemia. This mutation was excluded from analysis.

^b^Patient #8 had a bone marrow biopsy performed at an outside facility and was not available to review the morphology at the time of progression.

Of the remaining 14 patients with mutations, none progressed to an MN at a median follow-up of 24.9 months (range: 0.1−82.7). Ten patients had one mutation (*ASXL1, U2AF1, TET2, SRSF2, SF3B1, ASXL1, TET2, PHF6*), one patient had two mutations (*ASXL1/ASXL1*), and three patients had three mutations (*ASXL1/RUNX1/SRSF2, TET2/U2AF1/ASXL1, JAK2/TET2/TET2*; Table 3). One patient (#4, Table 3) had subsequent NGS performed at 71.8 months since isolated del(20q) detection and showed disappearance of the initial low level truncating *ASXL1* mutations (p.R404* and p.Q512*), but with acquisition of a new frameshift ASXL1 mutation (p. G646Yfs*12) at 24.6% VAF. Both bone marrow examinations of this patient showed no diagnostic features of an MN. The follow-up intervals for the three patients (#2, 6, 12, Table 3) with three mutations were 40.9, 8.4 and 0.2 months, respectively.

**Table 3:** Genetic mutations in patients who did not progress to myeloid neoplasms.

No	Age/Sex	Indication for bone marrow biopsy (% primary disease involvement)	Cytogenetics at initial isolated del(20q) detection	Mutations detected; (VAF)	Time to final follow-up (months)
1	90/M	Anemia, thrombocytopenia	46,XY,del(20)(q11.2q13.3)[7]/45,X,-Y[4]/46,XY[9]	***ASXL1***: c.1934dup; p.Gly646Trpfs*12; (6%)	47.3
2	82/M	Pancytopenia	46,XY,del(20)(q11.2q13.3)[6]/46,XY[14]	***ASXL1***: c.2120_2130del;p.Thr707Asnfs*7; (21%) ***RUNX1***: c.1360 T > C;p.*454Argext*52; (18.3%) ***SRSF2***: c.284 C > G;p.Pro95Arg; (25.4%)	40.9
3	82/M	Pancytopenia with macrocytosis	46,XY,del(20)(q11.2q13.3)[8]/46,XY[12]	***U2AF1***: c.101 C > T;p.Ser34Phe; (28.4%)	27.0
4	70/M	Multiple myeloma follow-up (No plasma cell neoplasm)	46,XY,del(20)(q11.2q13.3)[2]/46,XY[28]	First NGS: ***ASXL1***: c.1210 C > T;p.Arg404*; (5.6%) ***ASXL1***: c.1534 C > T;p.Gln512*; (6.7%) Second NGS: (at 71.8 months from 1^st^ NGS) ***ASXL1***: c.1934dup; p.Gly646Trpfs*12; (24.6%)	82.7
5	61/M	Mantle cell lymphoma follow-up (No lymphoma present)	46,XY,del(20)(q11.2q13.1)[13]/46,XY[7]	***TET2***: c.1630C > T; p.Arg544*; (29.3%)	64.4
6	76/M	Pancytopenia	46,XY,del(20)(q11.2q13.1)[2]/45,X,-Y[8]/46,XY[10]	***TET2***: c.3014del; p.Lys1005Argfs*2; (14.2%) ***U2AF1***: c.101 C > T; p.Ser34Phe; (29.2%) ***ASXL1***: c.1934dup; p.Gly646Trpfs*12; (24.4%)	8.4
7	76/M	Thrombocytopenia and monoclonal gammopathy (Plasma cell neoplasm < 5%)	46,XY,del(20)(q11.2q13.3)[20]	***SRSF2***: c.284 C > T; p.Pro95Leu; (33.3%)	0.1
8	81/M	Monoclonal gammopathy (No plasma cell neoplasm present)	46,XY,del(20)(q11.2q13.3)[6]/46,XY[14]	***SF3B1***: c.1998G > C;p.Lys666Asn; (43.8%)	24.3
9	58/M	CLL (20% CLL)	46,XY,del(20)(q11.2q13.1)[9]/46,XY[11]	***SF3B1***: c.2098 A > C;p.Lys700Gln; (9.9%)	7.5
10	71/M	Staging for diffuse large B-cell lymphoma (No lymphoma present)	46,XY,del(20)(q11.2q13.3)[4]/46,XY[16]	***ASXL1***: c.1900_1922del;p.Glu635Argfs*15; (15%)	0.9
11	68/M	Diffuse large B-cell lymphoma follow-up (No lymphoma present)	46,XY,del(20)(q11.2q13.1)[20]	***TET2***: c.5059 C > T;p.Gln1687*; (39.9%)	25.6
12	85/M	High-grade B-cell lymphoma NOS follow-up (No lymphoma present)	46,XY,del(20)(q11.2q13.3)[6]/46,XY[14]	***JAK2***: c.1849G > T;p.Val617Phe; (47.5%) ***TET2***: c.3885del;p.Tyr1295*; (47.1%) ***TET2***: c.4790del;p.Phe1597Serfs*13; (49.6%)	0.2
13	51/F	Progressive thrombocytopenia status-post temozolomide therapy for leiomyosarcoma	46,XX,del(20)(q11.2q13.3)[4]/46,XX[16]	***DNMT3A***: c.2204 A > G;p.Tyr735Cys; (8.6%)	21.7
14	56/M	Multiple myeloma follow-up (10% plasma cell neoplasm)	46,XY,del(20)(q11.2q13.3)[6]/46,XY[14]	***PHF6***: c.322_323insA;p.Ala108Aspfs*4; (8.3%)	40.2

*CLL* chronic lymphocytic leukemia, *VAF* variant allele frequency.

Evaluation of the functional pathways of the identified mutations in patients with and without MN progression revealed that non-DTA epigenetic modifier/nonspliceosome mutations, including *TP53, CBL, IDH1, IDH2, PHF6, BCOR, PTPN11, RUNX1, JAK2*, occurred significantly more frequently in patients with MN progression (8 of 9) in comparison to patients without MN progression (3 of 14) (*P* = 0.003). However, no significant differences in the frequency of the mutations involving DTA epigenetic modifiers (*DNMT3A, TET2, ASXL1*) or spliceosome genes (*SF3B1, U2AF1, SRSF2*) among the two groups, with DTA epigenetic modifiers seen in 4/9 versus 9/14 patients (*P* = 0.42), and mutations involving spliceosome genes in 2/9 versus 6/14 patients (*P* = 0.40), respectively.

As *ASXL1* gene is located on chromosome 20q11.21, we examined whether del(20q) affects *ASXL*1 allele burden if an *ASXL1* mutation is identified. Six patients carried *ASXL1* mutation(s) in our cohort, but no relationship could be established regarding the *ASXL1* VAF and the percentage of del(20q) (Supplemental Fig. 1). This could be explained by the fact that the *ASXL1* gene is not necessarily deleted when del(20q) is encountered. The breakpoints of del(20q) are heterogeneous that could be located more towards centromere regions or more towards telomere regions^9^. Since *ASXL1* is located close to centromere regions, this gene may not be disturbed in a 20q deletion. This is supported by a study that evaluated chromosomal microarray in 30 MDS patients with del(20q), and found 2/3 of patients had intact *ASXL1* gene and only 1/3 of patients had partial/entire *ASXL1* deletion^10^.

At initial diagnosis of isolated del(20q) in the 23 patients with mutations, the average percentage of del(20q) was 36.4% (SD ± 28.9%). The mean Hb, ANC and Plt count were 12.2 g/dL (SD ± 1.8; range: 8.3−14.8), 3.5 × 10^9^/L (SD ± 2.3, range: 0.6−9.4) and 126.9 × 10^9^/L (SD ± 80.9, range: 32−392), respectively. Statistical significance was not demonstrated with respect to age, sex, Hb, ANC, Plt, % VAF, prior history of cytotoxic chemotherapy, or % del(20q) metaphases among those with progression (*n* = 9) versus those without progression (*n* = 14) to an MN (Supplemental Table 1).

For the 33 patients without mutation, none of them progressed to an MN at a median follow-up of 33.9 months (range: 0.1−159.1). Statistical significance was not demonstrated on comparing patients with mutation(s) (*n* = 23) versus those without mutation (*n* = 33) with respect to % del(20q) metaphases, age, sex, prior history of cytotoxic chemotherapy, and other laboratory variables including Hb, ANC, and Plt (Supplemental Table 2). Four of these 33 patients had NGS performed on a follow-up bone marrow examination at a median follow-up of 18.7 months (range: 12.1−63.8), and none acquired mutations.

**Fig. 3:**
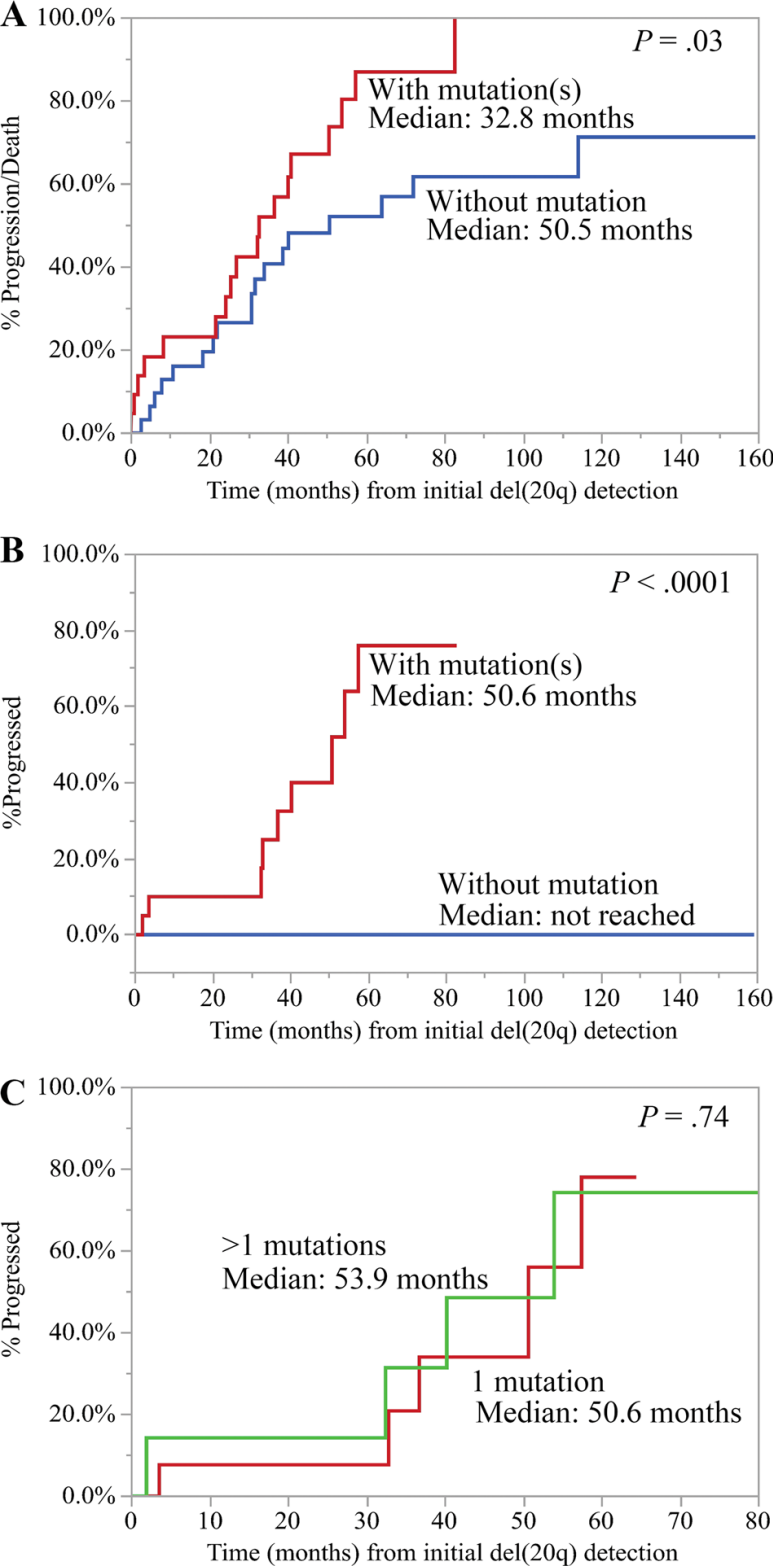
Kaplan−Meier analysis of time to progression among patients with isolated del(20q). **a** The time to progression and/or death among patients with mutation(s) (red) was 32.8 months and among those without mutation (blue) was 50.5 months (Log-rank *P* *=* 0.03). **b** Patients with mutation(s) (red) had a significantly higher risk of progression to a myeloid neoplasm in comparison to those without mutation (blue) (Log-rank *P* < 0.0001). **c** There was no significant difference in progression to a myeloid neoplasm among patients with one mutation (red) versus >1 mutations (green) (Log-rank *P* *=* 0.74).

Overall, at a median follow-up of 32.6 months (range: 0.1−159.1), 9 of the 56 patients progressed to MNs. On Kaplan−Meier survival analysis, the time to progression and/or death among patients with mutation(s) was 32.8 months and among those without mutation was 50.5 months (Log-rank *P* = 0.03, Fig. 3a). When the event of interest was progression to MN only, the presence of mutation(s) was associated with a significantly higher risk of disease progression into MNs (Log-rank *P* < 0.0001, Fig. 3b). At 5 years, at least 76% patients with mutation(s) progressed to a myeloid neoplasm (95% CI: 40.9−93.5, Fig. 3b). However, Kaplan−Meier analysis did not demonstrate statistical significance in the number of mutations (1 versus >1) associated with progression to MN (Log-rank *P* = 0.74, Fig. 3c).

## Discussion

Isolated del(20q) is associated with broad clinical presentations including neoplastic and nonneoplastic conditions, and can be seen in MNs such as MDS, MPN, MDS/MPN, and AML. Patients with MDS or MPN commonly have an indolent clinical course when del(20q) is the sole chromosomal alteration^11–13^, especially when del(20q) occurs at initial presentation^14^. However, the late occurrence of del(20q) in MDS, MPN or AML is usually associated with an unfavorable prognosis^14,15^. In addition to overt MNs, isolated del(20q) can be incidentally identified in patients without evident bone marrow morphologic features of involvement by an MN. Based on the presence or absence of cytopenia(s), these patients belong to either a nonneoplastic or preneoplastic category encompassing clonal hematopoiesis of indeterminate potential (CHIP) and clonal cytopenia(s) of undetermined significance^16,17^. The clinical outcome in patients with isolated del(20q) is generally indolent, but 10−25% ultimately develop MDS, MPN, MDS/MPN or AML^6,7,18^. The association of del(20q) with such a wide range of diseases and variable clinical outcomes clearly indicates the underlying genetic and biologic heterogeneity. It further implies that additional abnormalities (second hit) are required for the development of an MN in patients with unremarkable bone marrow morphology at the time of isolated del(20q) detection.

In our study, mutations were detected in 41.1% of isolated del(20q) patients, of whom 16.1% progressed into MNs during the course of follow-up. All progressed patients harbored one or more pathogenic mutations at the time of isolated del(20q) diagnosis and the presence of mutation is a statistically significant risk factor for MN progression. Additionally, among the 23 patients harboring mutations, the presence of non-DTA epigenetic modifier/spliceosome mutations were strongly associated with MN progression (8/9 versus 3/14, *P* = 0.003), whereas mutations occurring in the DTA epigenetic modifiers and spliceosome genes showed no statistically significant differences in patient with and without progression. The mutations involving non-DTA epigenetic modifiers include *IDH1, IDH2* and *BCOR*, kinases *CBL, PTPN11* and *JAK2*, tumor suppressors *TP53 and PHF6*, and transcription factor *RUNX1*. No statistically significant association with MN progression was observed in the percentages of VAF or del(20q), patient age, gender, Hb, ANC, Plt, or prior history of chemotherapy.

Certain mutations may play a critical role in the pathogenesis, evolution and disease progression of MNs. The strong association of presence of non-DTA epigenetic modifier/nonspliceosome mutations with MN progression observed in our study (8/9 verse 3/14, *P* = 0.003) was also in keeping with the documented pathogenic role of mutations in signaling pathway kinases, transcription factors, tumor suppressors and non-DTA epigenetic modifiers. They have been shown to be associated with disease progression in MDS and cooperate with earlier events of DTA epigenetic modifier and spliceosome mutations to drive disease progression. In contrast, mutations of epigenetic modifiers *DNMT3A, TET2* and *ASXL1* (DTA mutations) are commonly seen in MNs, they are also relatively frequently mutated in healthy aging individuals and represent the most common mutations of CHIP^16,19^. Isolated mutations in *DNMT3A, TET2* or *ASXL1*, particularly those with a low VAF, showed a low positive predictive value for MN development^20^. Detection of DTA mutations in AML patients in complete remission is also not associated with an increased risk of relapse, supporting the premalignant nature of these mutations^21^. Our findings were consistent with previous studies showing no differences in DTA mutation frequency among isolated del(20q) patients with or without MN progression (4/9 verse 9/14, *P* = 0.42). Of note, *ASXL1* is located on chromosome 20q11.21, and *ASXL1* gene is partially/completely deleted in approximately 1/3 of patients with del(20q)^10^. Therefore, del(20q) sometimes “pheno-copy” *ASXL1* mutation to give rise to clonal hematopoiesis. This study broadens the current concept of clonal hematopoiesis and implies that clonal hematopoiesis is resulted from not only the genetic mutation(s), but also cytogenetic abnormality.

Some patterns of mutations identified as determining factors for the progression to MN in patients with unexplained cytopenia(s)^20^ were not detected in our cohort. For instance, it has been shown that mutations in the RNA spliceosome factors (*SF3B1, SRSF2*, *U2AF1*, and *ZRSR2*), or ≥2 mutations, or those with mutation associated with high VAF, had a high positive predictive value for the development of an MN in patients with unexplained cytopenia(s)^20^. However, we did not find a clear association of spliceosome mutations, number of mutations or %VAF with MN progression in isolated del(20q) patients. The observed discrepancies with previous reports may be attributable to the presence of the distinct genetic background of del(20q), the relatively small sample size and the relatively short follow-up for some patients in our study cohort. Moreover, factors other than mutations may contribute to the development of an MN, such as immune dysregulation, chromosomal instability and detrimental marrow microenvironment^22^. We did not encounter *ZRSR2* mutations in our patients, conforming to the rarity and exclusion of *ZRSR2* from the list of CHIP genes which include other more common spliceosome genes *SF3B1, U2AF1 and SRSF2* (ref. ^23^).

Interestingly, six patients had a decrease/disappearance of del(20q) during disease progression in our cohort. Three patients gained complex cytogenetics, two gained an additional mutation in a different gene and one had increased VAF of the pre-existing mutations. This finding indicates the survival/proliferative disadvantage of del(20q) clone compared to other aggressive clones. The presence of a 20q deletion may be an initial risk factor that provides milieu for the growth of other aggressive clones. Our study reveals the genetic changes in MNs are heterogeneous and it demonstrates dynamics of clonal evolution in the progression of a myeloid neoplasm.

In our cohort, all 33 patients without mutations did not develop an MN. This finding indicates the lack of mutations had a low risk of MN progression in patients with isolated del(20q) and it further implies the presence of sole del(20q) is insufficient for the development of an MN. Consistent with our finding, the absence of mutations is a negative predictor for MN progression among patients with unexplained cytopenia^20,24^. Additionally, the advent of large-scale sequencing analyses have shown the vast majority of MDS patients are associated with somatic mutations^25–27^, providing further evidence of a high negative predictive value for the lack of identifiable mutations to MN development.

In summary, at the time of isolated del(20q) detection, patients without mutations had a very low risk for progression to an MN, while approximately one-third of those patients with mutations ultimately developed an MN. The subsequent development of MNs was significantly associated with the presence of mutation(s), but not with the number of mutations, %VAF, % del(20q), age, gender, Hb, ANC, Plt, or prior history of chemotherapy. The presence of non-*DTA* epigenetic modifier/nonspliceosome mutations was significantly associated with MN progression in our study cohort. Overall, these data support genetic mutation analysis as a valuable complement to the current diagnostic evaluation of patients with isolated del(20q), which may facilitate appropriate risk stratification and guide treatment decisions.

## Supplementary information

Supplemental Figure 1

Supplemental Table 1

Supplemental Table 2
