# Supplementary material for: The significance of genetic mutations and their prognostic impact on patients with incidental finding of isolated del(20q) in bone marrow without morphologic evidence of a myeloid neoplasm
Source: Blood Cancer J. 2020 Jan 23;10(1):7. doi: 10.1038/s41408-020-0275-8 (PMC6978416; doi:10.1038/s41408-020-0275-8)
Supplement: Supplementary file 1 — Supplemental Figure 1 [file 41408_2020_275_MOESM1_ESM.docx]

**Supplemental Figure 1:** Graphical representation demonstrating a random association between % del(20q) metaphases and % variant allele frequency (VAF) in patients with ASXL1 mutation.

**
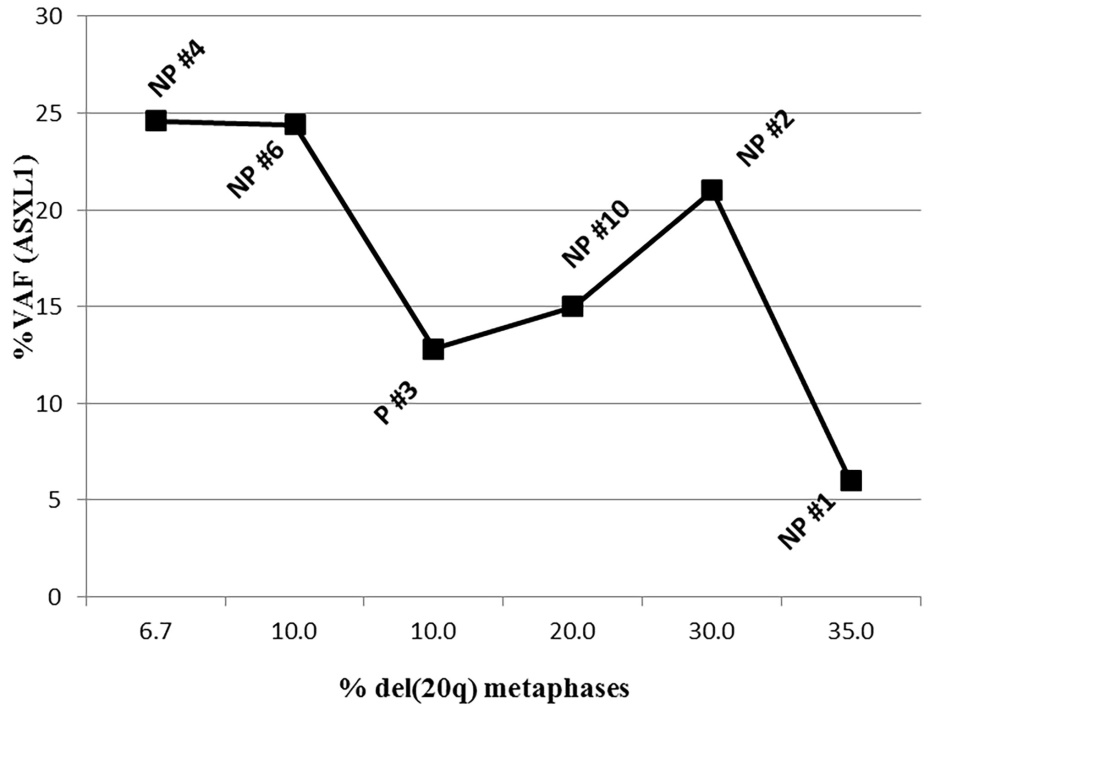
**

**Abbreviations**: NP: Not Progressed-Patients with ASXL1 mutation(s) and without progression to a myeloid neoplasm (table 3).

Patient #4 (NP# 4) had two different mutations in the ASXL1 gene and the mutation with the higher VAF was considered for plotting the graph.

P: Progressed-Patients with ASXL1 mutation(s) and with progression to a myeloid neoplasm (table 2)
