# Supplementary material for: The significance of genetic mutations and their prognostic impact on patients with incidental finding of isolated del(20q) in bone marrow without morphologic evidence of a myeloid neoplasm
Source: Blood Cancer J. 2020 Jan 23;10(1):7. doi: 10.1038/s41408-020-0275-8 (PMC6978416; doi:10.1038/s41408-020-0275-8)
Supplement: Supplementary file 2 — Supplemental Table 1 [file 41408_2020_275_MOESM2_ESM.docx]

**Supplemental Table 1: Comparison of clinical and laboratory features of patients with isolated del(20q) among those with mutation(s) and progression versus those with mutation(s) and without progression to a myeloid neoplasm**

| **Variable** | **With Mutation and progression**  **(n=9)** | **With Mutation and without progression**  **(n=14)** | ***P* value** |
| --- | --- | --- | --- |
| Age, years | Mean: 69.4 (SD ±9.8)  (range: 55-88) | Mean: 71.6 (SD ±12)  (range: 51-90) | .65 |
| Sex | Male: 6 (66.7%)  Female: 3 (33.3%) | Male: 13 (92.9%)  Female: 1 (7.1%) | .26 |
| Hemoglobin, g/dL | Mean: 12.3 (SD ±1.6)  (range:9.3-14.7) | Mean: 12.1 (SD ±1.9)  (range: 8.3-14.8) | .80 |
| Absolute neutrophil count, x10^9^/L | Mean: 3.2 (SD ±2.5)  (range: 0.6-8) | Mean: 3.7 (SD ±2.3)  (range: 0.9-9.4) | .63 |
| Platelet count, x10^9^/L | Mean: 156.9 (SD ±108.7)  (range: 61-392) | Mean: 107.7 (SD ±52.7)  (range: 32-207) | .16 |
| %VAF | Mean: 28.6 (SD ±17.5)  (range: 5.2-53.4) | Mean: 22.9 (SD ±14.6)  (range: 6-48.1) | .40 |
| % Del(20q) | Mean: 30.6 (SD ±29.2)  (range: 10-100) | Mean: 40.1 (SD ±29.2)  (range: 6.7-100) | .45 |
| Cytotoxic chemotherapy | 7 (77.8%) | 9 (64.3%) | .66 |
