# Supplementary material for: The significance of genetic mutations and their prognostic impact on patients with incidental finding of isolated del(20q) in bone marrow without morphologic evidence of a myeloid neoplasm
Source: Blood Cancer J. 2020 Jan 23;10(1):7. doi: 10.1038/s41408-020-0275-8 (PMC6978416; doi:10.1038/s41408-020-0275-8)
Supplement: Supplementary file 3 — Supplemental Table 2 [file 41408_2020_275_MOESM3_ESM.docx]

**Supplemental Table 2: Comparison of clinical and laboratory features of patients with isolated del(20q) among those with mutation(s) versus those without mutation**

| **Variable** | **With Mutation(s)**  **(n=23)** | **Without Mutation**  **(n=33)** | ***P* value** |
| --- | --- | --- | --- |
| Age, years | Mean: 70.7 (SD ±11.1)  (range: 51-90) | Mean: 65.8 (SD ±10.7)  (range: 44-83) | .10 |
| Sex | Male: 19 (82.6%)  Female: 4 (17.4%) | Male: 23 (69.7%)  Female: 10 (30.3%) | .35 |
| Hemoglobin, g/dL | Mean: 12.2 (SD ±1.8)  (range: 8.3 -14.8) | Mean: 11.4 (SD ±1.5)  (range: 8.5-15.1) | .08 |
| Absolute neutrophil count, x10^9^/L | Mean: 3.5 (SD ±2.3)  (range: 0.6-9.4) | Mean: 2.9 (SD ±1.2)  (range: 0.8-5.5) | .21 |
| Platelet count, x10^9^/L | Mean: 126.9 (SD ±80.9)  (range: 32-392) | Mean: 163.7 (SD ±84.7)  (range: 16-385) | .11 |
| % Del(20q) | Mean: 36.4 (SD ±28.9)  (range: 6.7-100) | Mean: 39.8 (SD ±29.8)  (range: 6.7-100) | .67 |
| Cytotoxic chemotherapy | 16 (69.6%) | 22 (66.7%) | .82 |
